# Neurocognitive features of children and adolescents with different levels of multitasking

**DOI:** 10.1192/j.eurpsy.2022.699

**Published:** 2022-09-01

**Authors:** G. Soldatova, A. Vishneva, A. Koshevaya

**Affiliations:** 1 Lomonosov Moscow State University, Faculty Of Psychology, Moscow, Russian Federation; 2 Center for speech pathology and neurorehabilitation, Clinical Psychology, Moscow, Russian Federation

**Keywords:** Adolescents, neuropsychological profile, multitasking, Children

## Abstract

**Introduction:**

The spread of media multitasking in the modern world determines researcher’s interest in studying the neurocognitive development features of children who strive to act in this mode since childhood (Minear et al., 2013; Uncapher et al., 2016).

**Objectives:**

The aim is to study neuropsychological profiles of children and adolescents with single-tasking and multitasking.

**Methods:**

Quasi-experiment was conducted among 154 children of three age groups (7-10; 11-13; 14-16) and included simultaneous tasks performance on a computer and a smartphone. Neuropsychological indicators were studied (Akhutina, 2016): programming and control, serial organization, visual and auditory-speech memory, neurodynamics. The behavior social modeling and executive functions were studied with WISC (Information and Comprehension subtests) and Dots-test (Akhutina et al., 2017). The multitaskers groups were identified according to the number of returns to tasks: single-taskers (42.9%), single-taskers with multitasking elements (1-2 returns) (40.9%), multitaskers (3 and more returns) (16.2%).

**Results:**

The number of multitaskers increased by adolescence. In children aged 7-10 single-taskers were more productive than multitaskers in programming, switchability, the volume of auditory-speech and visual memory, neurodynamics. They performed Dots-test faster. At the age of 11-13, multitaskers had higher scores on the Comprehension subtest and higher verbal activity when composing a story. Regulation and switchability errors in multitaskers aged 11-13 were episodic. At the age of 14-16 multitaskers were more productive in some parameters of switchability.

**Conclusions:**

In primary school single-taskers have a neurocognitive advantage, but by adolescence differences are leveled, and in some parameters multitaskers are ahead of single-taskers. The study was funded by RFBR, project No. 19-29-14181.

**Disclosure:**

The reported study was funded by RFBR, project No. 19-29-14181.

